# Lessons learned: Retrospective assessment of outcomes and management of patients with advanced HIV disease in a semi-urban polyclinic in Epworth, Zimbabwe

**DOI:** 10.1371/journal.pone.0214739

**Published:** 2019-04-10

**Authors:** Simon Blankley, Tadele Gashu, Bilal Ahmad, Abi kebra Belaye, Lucia Ringtho, Anita Mesic, Simukai Zizhou, Esther C. Casas

**Affiliations:** 1 Médecins Sans Frontières/Doctors Without Borders, Harare, Zimbabwe; 2 Médecins Sans Frontières/Doctors Without Borders, Berlin, Germany; 3 Médecins Sans Frontières/Doctors Without Borders, Amsterdam, Netherlands; 4 Provincial Medical Directorate, Ministry of Health and Child Care, Marondera, Zimbabwe; University of Pretoria, SOUTH AFRICA

## Abstract

**Introduction:**

HIV continues to be one of the leading causes of infectious death worldwide and presentation with advanced HIV disease is associated with increased morbidity and mortality. Recommendations for the management of advanced HIV disease include prompt screening and treatment of opportunistic infections, rapid initiation of ART and intensified adherence support. We present treatment outcomes of a cohort of patients presenting with advanced HIV disease in a semi-urban Zimbabwean polyclinic.

**Methods:**

Retrospective cohort analysis of adult patients enrolled for care at Epworth polyclinic, Zimbabwe between 2007 and end June 2016. Treatment outcomes at 6 and 12 months were recorded. Multivariate logistical regression analysis was undertaken to identify risk factors for presentation with advanced HIV Disease (CD4 count less than 200 cells/mm^3^ or WHO stage 3 or 4) and risks for attrition at 12 months.

**Results:**

16,007 anti-retroviral therapy naive adult patients were included in the final analysis, 47.4% of whom presented with advanced HIV disease. Patients presenting with advanced HIV disease had a higher mortality rate at 12 months following enrollment compared to early stage patients (5.11% vs 0.45%). Introduction of a package of differentiated care for patients with a CD4 count of less than 100 cells/mm^3^ resulted in diagnosis of cryptococcal antigenaemia in 7% of patients and a significant increase in the diagnosis of TB, although there was no significant difference in attrition at 6 or 12 months for these patients compared to those enrolled prior to the introduction of the differentiated care.

**Conclusions:**

The burden of advanced HIV disease remained high over the study period in this semi-urban polyclinic in Zimbabwe. Introduction of a package of differentiated care for those with advanced HIV disease increased the diagnosis of opportunistic infections and represents a model of care which can be replicated in other polyclinics in the resource constrained Zimbabwean context.

## Introduction

HIV continues to be one of the leading causes of infectious death worldwide, in 2016 it is estimated that 36.7 million people were living with HIV and infection with HIV was responsible for 1 million deaths worldwide [1,2]. Zimbabwe, an Eastern and Southern African country has a high burden of HIV infection, in 2016 an estimated 1.3 million people were living with HIV infection in Zimbabwe[2].

Presentation with advanced HIV disease (CD4 count less than 200 cells/mm^3^ or WHO stage 3 or 4) remains high in 2016 with 29% of those enrolled on treatment worldwide having advanced HIV disease[2]. Presentation with advanced HIV disease is associated with increased morbidity and mortality, increased risk of onward transmission as well as higher societal healthcare costs [3–6]. Patients in developing countries tend to first present with lower CD4 counts compared to developed countries [7,8]. Many potential risk factors for presentation with advanced HIV disease have been identified which include male sex, increasing age, low socio-economic status, poor social support and fear of stigma [6,9–13].

In 2017 new guidance on the management of patients with advanced HIV disease was issued by the WHO and include recommendations for prompt screening and treatment of opportunistic infections [13]. Testing for Cryptococcal antigenaemia in patients with CD4 counts less than 100cells/mm^3^ has been shown to reduce mortality from cryptococcal meningitis and cryptococcal immune reconstitution inflammatory syndrome [14,15]. Testing and treating for Cryptococcal antigenemia is likely to be cost effective and has already been adopted at the national level in some countries, including Zimbabwe where since December 2016 it along with screening for tuberculosis (TB) has been recommended for all patients presenting with CD4 count less than 100cells/mm^3^, it is possible that testing patients with a CD4 count less than 200 cells/mm^3^ may yield additional cases [13,16–18].

In this study we aim to describe the burden of advanced HIV disease and the 6 and 12 month outcomes of ART naïve patients following enrollment at the Epworth polyclinic, Zimbabwe between 2007 and 2016. In addition, we aim to describe and assess if the addition of an enhanced package of care for those with CD4 counts less than 100 cells/mm^3^, including reflex testing for Cryptococcal antigenemia and screening for Tuberculosis (TB) had any impact on attrition rates (attrition in the cohort is defined as those patients lost to follow up or known to have died from any cause combined).

## Methods

A descriptive retrospective cohort study using routine data collected under programmatic conditions in Epworth polyclinic, Zimbabwe for clients enrolled for HIV care between 1^st^ January 2007 and 30th June 2016, with follow up data on patients collected until 30^th^ June 2017.

The Epworth polyclinic is a Zimbabwean Ministry of Health and Child Care (MoHCC) polyclinic supported by Médecins Sans Frontières (MSF). Epworth is a high density town (population 167,462, 2012 census [19]) on the outskirts of the capital Harare. The HIV clinic at Epworth polyclinic was established in 2006 and is in principle a nurse (both MSF and MoHCC nurses) led service with support available on site from both MSF and MoHCC doctors.

All adult patients (aged 19 years or older) that were ART naïve and enrolled for HIV care at the clinic were included in this analysis, patients that were transferred-in already on ART, did not have a first visit CD4 count, or were missing a first visit WHO staging that prevented classification into a stage of presentation were excluded. Advanced HIV disease used the recent consensus definition of presentation with a CD4 count of less than 200 cells/mm^3^ or at WHO stage 3 or 4, other patients were labelled as early presentation [13,20].

There have been changes to the standard of care that HIV patients receive at the Epworth HIV clinic during the study period, which relate both to changes in HIV policy and guidelines at the national or clinic level, and following international ART recommendations. Over time the differentiated care offered to patients presenting with advanced disease has changed, with the introduction of different components at different times, sometimes on an informal ad-hoc basis. Such components included a daycare unit (since 2014) and cryptococcal antigen testing (from 2015). By February 2015 a more formalized package of differentiated care was offered for patients presenting with advanced HIV disease and a CD4 count less than 100cells/mm^3^ which included being seen in a doctor led day clinic where more extensive investigations would be routinely ordered as a package of care including serum cryptococcal antigen (CRAG) testing and sputum GeneXpert testing. Also, ultrasound, referral for chest x-ray and lumbar puncture were available if clinically indicated. If the serum CRAG was positive and there was no clinical evidence of cryptococcal meningitis then a lumbar puncture was undertaken to determine the cerebrospinal fluid (CSF) CRAG status. All patients were given co-trimoxazole prophylaxis (unless documented allergy, in which case Dapsone was used), patients were not routinely given other prophylactic antibiotics, although patients if unwell were assessed for and treated for bacterial infections as indicated. Daycare ambulatory treatment for these patients was also available at the clinic including but not limited to anti-fungal therapy, antibiotics and intravenous fluids. Patients with confirmed Cryptococcal meningitis were referred for inpatient management at a local government hospital.

The data for this analysis was collected from the paper based clinical records which was contemporaneously entered into an electronic database. A further round of data entry was undertaken between June 2017 and August 2017 to ensure that the research database was as complete as possible. Data was exported, merged and cleaned, checks on data accuracy and plausibility were carried out by the clinic data team and the research team including verifying a subset of database data against the original paper based record. Data was assessed to determine if data was missing and if it was missing at random.

Statistical analyses were undertaken in Rstudio (V 1.0.153), comparative statistical testing was undertaken using either Chi Square or exact Fisher testing. For comparison of numerical data it was first assessed for normalcy of distribution. Normally distributed data was then compared using either t-test or ANOVA, data not normally distributed was compared using Mann-Whitney testing or Kruskal-Wallis statistical tests. Two multivariate models were constructed, Risk factors for presentation with advanced HIV disease used linear regression modelling constructed using basic demographic variables. A cox-proportionate hazard models regression model was constructed using outcome data, time to outcome, demographic characteristics, tuberculosis diagnosis, enrolment CD4 count, ART regimen and Year of enrolment. Only those factors significant in univariate analysis were included in the multivariate model.

Ethical approval for this study was obtained from the Medical Research Council of Zimbabwe (REF: MRCZ/E/169). The study met the criteria for analysis of routinely collected program data of the MSF independent Ethics Review Board and individual consent from patients was not sought [21].

## Results

**Fig 1:**
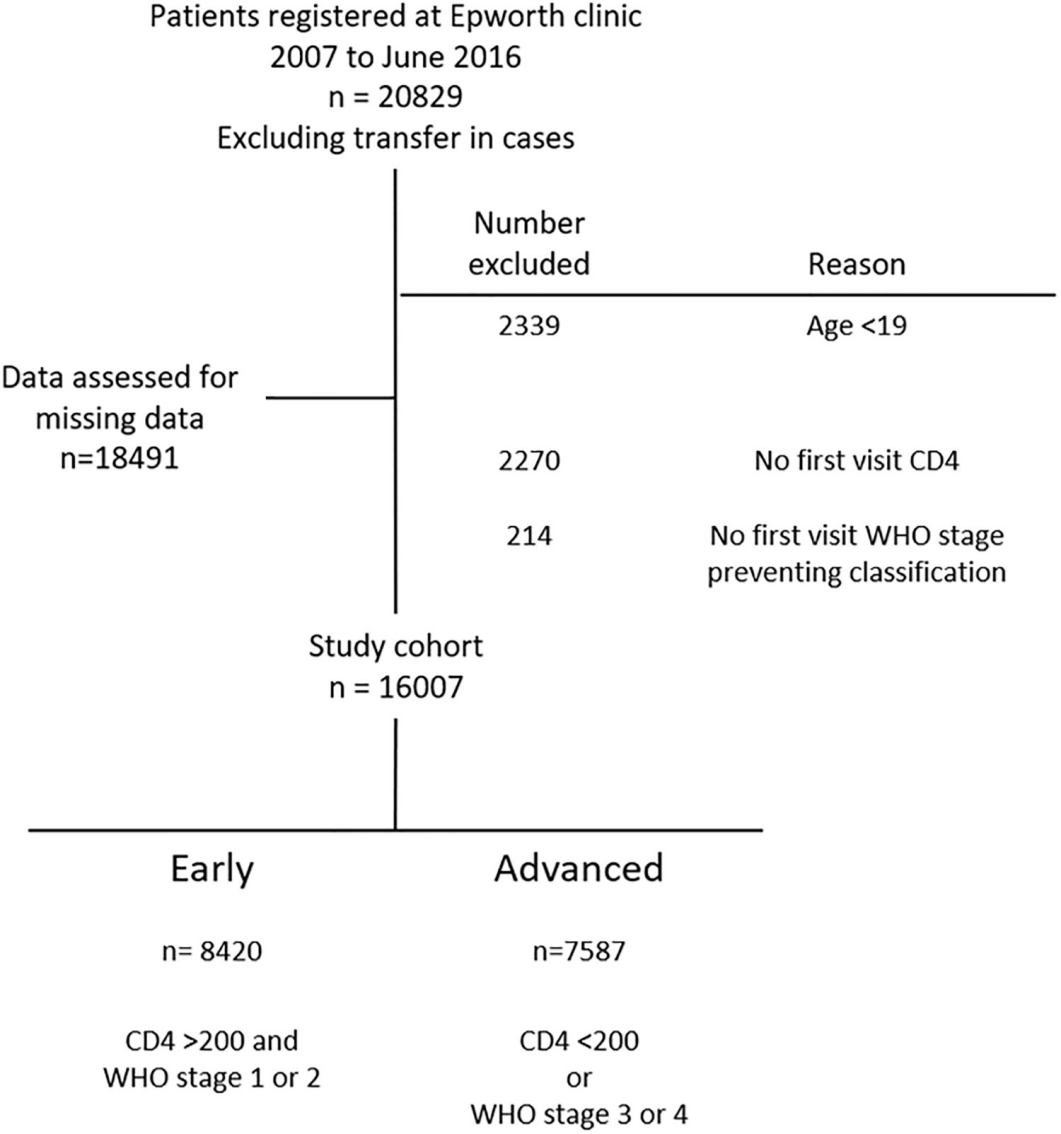
Enrollment in to the cohort.

A total of 20,829 ART naïve HIV infected patients were enrolled at the Epworth Polyclinic between January 1^st^ 2007 and the 30^th^ June 2016. Transfer-in cases were excluded from the database by the data team based on their ART number and a further 2,338 were excluded from further analysis as they were aged under 19 (Fig 1).

Data was then assessed for missing data in demographic details as well as first visit CD4 count and first visit WHO stage. There was complete data for age, sex and minimally missing data (<1%) for entry to clinic and marital status. A large percentage of data was missing for educational status and first visit body mass index (BMI), first visit CD4 count was missing in 12.3% of patients.

Patients were then grouped by stage of presentation, 2,484 were excluded as they either lacked a documented first visit CD4 count or WHO stage preventing classification, the remaining cohort of 16,007 was divided into those presenting with or without advanced HIV disease with 7,587 (47.4%) presenting with advanced HIV disease (Fig 1).

The median age of the cohort was 33 years old (IQR 28–39), with patients presenting with advanced HIV disease presenting at a significantly older median age compared to early stage patients (35 vs 31 years old, Table 1). Most of the patients enrolled into the cohort were female (62.6% of all those enrolled), males were significantly more likely to present with advanced HIV disease (45.6% of males presenting with advanced HIV disease compared to 29.9% of females, Table 1). Patients at an early stage compared were more likely to have been enrolled via PMTCT compared to with advanced HIV disease (9.39% compared to 3.56%, Table 1).

**Table 1:** Demographics of cohort.

	Overall n = 16007	Early n = 8420	Advanced n = 7587	p value	n.
Age (IQR)	33.0 (28.0–39.0)	31.0 (26.0–37.0)	35.0 (29.0–41.0)	<0.001	16007
Sex:				<0.001	16007
Male	5980 (37.4%)	2517 (29.9%)	3463 (45.6%)		
Female	10027 (62.6%)	5903 (70.1%)	4124 (54.4%)		
BMI:				<0.001	12845
<18	2521 (19.6%)	578 (8.98%)	1943 (30.3%)		
18–24	8097 (63.0%)	4261 (66.2%)	3836 (59.8%)		
>24	2227 (17.3%)	1594 (24.8%)	633 (9.87%)		
Point of entry:				<0.001	15924
VCT	13153 (82.6%)	6728 (80.4%)	6425 (85.0%)		
Health Care Facility	1617 (10.2%)	777 (9.29%)	840 (11.1%)		
PMTCT	1054 (6.62%)	785 (9.39%)	269 (3.56%)		
Other	100 (0.63%)	74 (0.88%)	26 (0.34%)		
Marital status:				<0.001	15995
Married	10799 (67.5%)	5993 (71.2%)	4806 (63.4%)		
Divorced	1880 (11.8%)	953 (11.3%)	927 (12.2%)		
Single	1093 (6.83%)	511 (6.07%)	582 (7.68%)		
Widowed	2223 (13.9%)	957 (11.4%)	1266 (16.7%)		
Education level:				0.215	7508
Primary	1991 (26.5%)	1050 (26.4%)	941 (26.7%)		
Secondary	5412 (72.1%)	2883 (72.4%)	2529 (71.7%)		
Tertiary	105 (1.40%)	47 (1.18%)	58 (1.64%)		
Profession:				<0.001	14893
Employed	3574 (24.0%)	1611 (21.2%)	1963 (27.0%)		
Unemployed	9998 (67.1%)	5434 (71.4%)	4564 (62.7%)		
Other	1321 (8.87%)	566 (7.44%)	755 (10.4%)		

Table of basic demographic characteristics of overall cohort and by stage of presentation. Number and percentage given for each category, except age where median value and IQR range is given. Chi squared test or Mann-Whitney test used to test for significance of difference between stages of presentation. VCT: Voluntary counselling and testing, PMTCT: Prevention of mother to child transmission.

**Fig 2:**
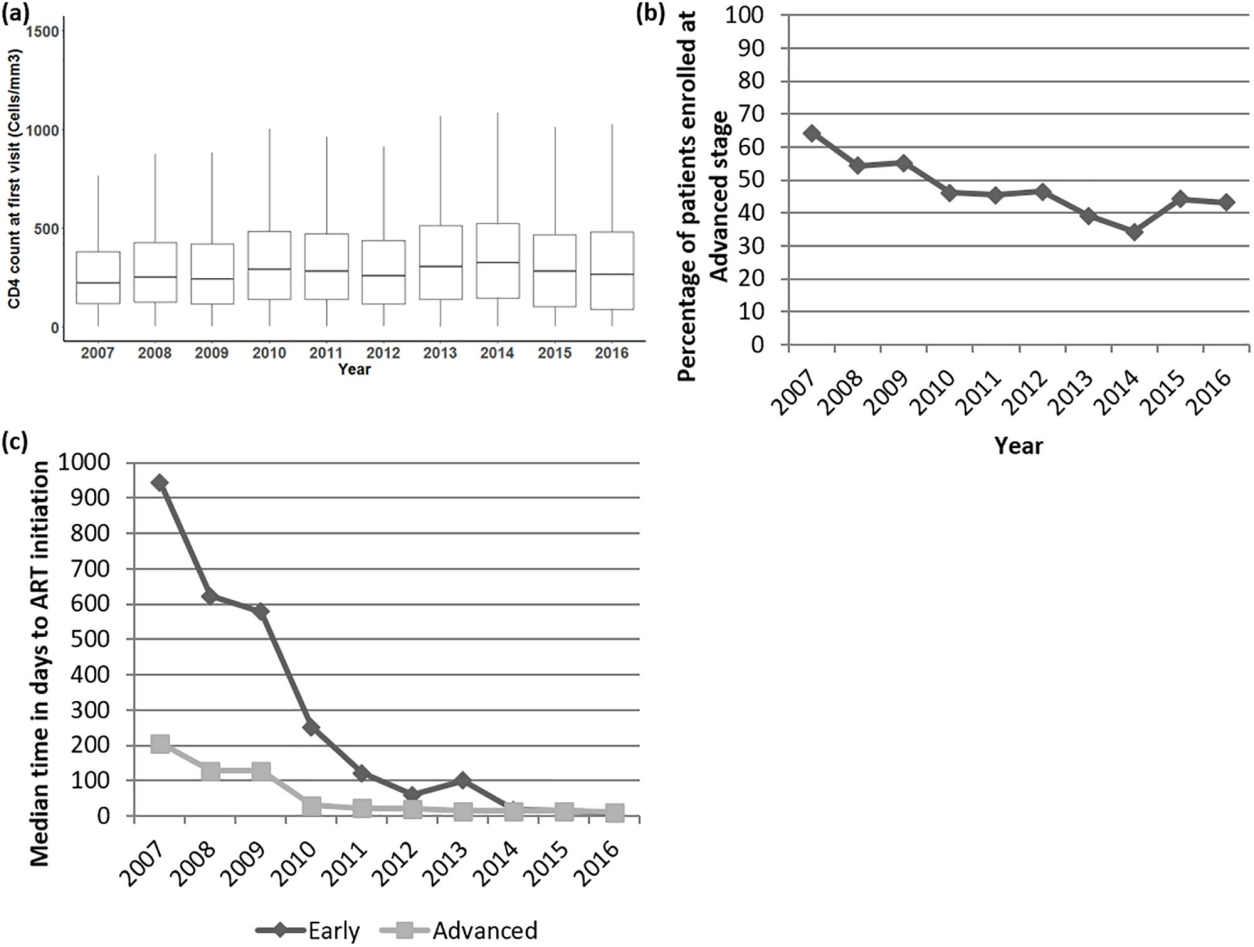
Changes over time in enrollment and stage of presentation at first visit. **(A)** Boxplot of median CD4 count at first visit (n = 16007, horizontal line within box representing median value, box lower bounds at 25^th^ centile, upper bounds of box at 75^th^ centile, whiskers represent the largest or smallest value up to a maximum of 1.5x the inter-quartile range with further outliers not plotted), statistical test to determine difference in CD4 count at first visit between years; Kruskal-Wallis p<0.001. **(B)** Percentage of patients enrolled per year at an advanced stage. Statistical test to determine difference; Exact Fisher test, p<0.001. **(C)** Median time in days to anti-retroviral initiation in those that started anti-retroviral therapy stratified disease status at first visit (n = 11853). Statistical test of significance; Kruskal-Wallis, p<0.001.

There was a statistically significant although small increase in median CD4 count at first visit from a median of 222 cells/mm^3^ (mean: 281 cells/mm^3^) in 2007 to a median of 264cells/mm^3^ (mean: 338 cells/mm^3^) in 2016, representing an average increase of median CD4 count of 4.8 cells/mm^3^ per year (mean increase: 6.4 cells/mm^3^ per year, Fig 2A). The percentage of ART naïve patients enrolling into the cohort with advanced HIV disease decreased year on year, from 60% of the cohort enrolled in 2007 to just over 40% of those enrolled in 2016 (Fig 2B). The median time to ART initiation in those with both advanced HIV disease and early stage disease has significantly decreased over time, with a median time of 128 days in 2008 for those with advanced HIV disease compared to a median of 11 days in 2016 (Fig 2C).

Multivariate logistical regression analysis was undertaken to identify risk factors for presentation with advanced HIV disease which identified male sex (Adjusted odds ratio (OR) 1.15), older age (Adjusted OR 1.23), referral from a health care facility and not being married were all associated with a higher risk of late presentation (Table 2).

**Table 2:** Multivariate analysis for risk factors for advanced disease at first presentation.

	Crude			Adjusted		
	OR	95% CI	p value	AOR	95% CI	p value
Sex:						
Female	Ref.	Ref.	Ref.	Ref.	Ref.	Ref.
Male	1.97	(1.85–2.10)	<0.001	1.1497	(1.1295–1.1704)	<0.001
Age:						
19–24	Ref.	Ref.	Ref.	Ref.	Ref.	Ref.
24–40	2.23	(1.98–2.51)	<0.001	1.1466	(1.1149–1.1792)	<0.001
40–60	3.55	(3.12–4.05)	<0.001	1.2258	(1.187–1.266)	<0.001
>60	3.81	(2.94–4.94)	<0.001	1.2305	(1.1537–1.3125)	<0.001
Point of entry:						
VCT	Ref.	Ref.	Ref.	Ref.	Ref.	Ref.
Health Care Facility	1.13	(1.02–1.26)	0.019	1.0733	(1.0432–1.1043)	<0.001
PMTCT	0.36	(0.31–0.41)	<0.001	0.8733	(0.8455–0.9021)	<0.001
Other	0.37	(0.23–0.57)	<0.001	0.8798	(0.7797–0.9928)	0.0378
Marital Status:						
Married	Ref.	Ref.	Ref.	Ref.	Ref.	Ref.
Divorced	1.21	(1.10–1.34)	<0.001	1.0714	(1.0448–1.0988)	<0.001
Single	1.42	(1.25–1.61)	<0.001	1.1246	(1.0891–1.1613)	<0.001
Widowed	1.65	(1.50–1.81)	<0.001	1.128	(1.101–1.1557)	<0.001
Profession:						
Employed	Ref.	Ref.	Ref.	Ref.	Ref.	Ref.
Unemployed	0.69	(0.64–0.74)	<0.001	0.9449	(0.9273–0.9629)	<0.001
Other	1.09	(0.96–1.24)	0.164	0.9962	(0.966–1.0273)	0.8078

Complete case analysis (n = 14839), identifying risk factors for advanced disease at first presentation (CD4 less than 200 cells/mm^3^ or WHO stage 3 or 4). Model constructed using basic demographic details (Table 1) excepting BMI and education level for which data was missing in a large number of patients. OR: Crude odds ratio, AOR: Adjusted Odds ratio.

Defined outcomes at 6 and 12 months were available for all patients (n = 16,007, Table 3). Overall at 6 months 1.9% of the cohort had died and 12.5% of the cohort was lost to follow up, giving an attrition rate (Lost to follow up and deaths combined) of 14.4% at 6 months and 23% at 12 months (Table 3). Attrition at both 6 and 12 months was significantly higher in those presenting with advanced HIV disease compared to those presenting at an early stage (Table 3).

**Table 3:** Treatment outcomes of cohort and stratified by stage of presentation.

	Overall n = 16007	Early n = 8420	Advanced n = 7587	p.value	n.
Status at 6 months:				<0.001	16007
Active on follow up	13538 (84.6%)	7297 (86.7%)	6241 (82.3%)		
Died	298 (1.86%)	14 (0.17%)	284 (3.74%)		
Lost to follow up	1999 (12.5%)	1046 (12.4%)	953 (12.6%)		
Transferred out	172 (1.07%)	63 (0.75%)	109 (1.44%)		
Status at 12 months:				<0.001	16007
Active on follow up	11984 (74.9%)	6433 (76.4%)	5551 (73.2%)		
Died	426 (2.66%)	38 (0.45%)	388 (5.11%)		
Lost to follow up	3270 (20.4%)	1823 (21.7%)	1447 (19.1%)		
Transferred out	327 (2.04%)	126 (1.50%)	201 (2.65%)		

Active on follow up: The patient remains in the cohort and is known to be alive at the timepoint. Lost to follow up: Patient has missed their last appointment by at least 2 months and no contact has been made with the patient, Transferred out: The patient is confirmed to have been transferred to another treatment facility for ongoing care.

Statistical testing between stages of presentation: Chi squared test, p<0.001 for both 6 and 12 months.

A cox-proportionate hazard model was constructed to identify risk factors for attrition in ART naïve patients presenting with advanced HIV disease over the first 12 months following enrollment into the cohort. The model took into account basic demographic details as well as year of enrollment, CD4 count at enrollment, initial anti-retroviral regimen as well the timing of a diagnosis of TB. The model identified single marital status was associated with a significantly higher risk (adjusted hazard ratio 1.17) of attrition over 12 months as was a CD4 count less than 200 cells/mm^3^, with the risk increasing the lower the CD4 count (Table 4). Regimens containing Stavudine were also associated with an increased risk of attrition over 12 months as was enrollment after 2007, Table 4).

**Table 4:** Multivariate analysis for risks of attrition over 12 months of follow up for patients presenting at an advanced stage.

	Crude			Adjusted		
	HR	CI 95%	p value	HR	CI 95%	p value
Age:						
19–24	ref.	ref.	ref.	ref.	ref.	ref.
24–40	0.79	(0.66–0.96)	0.0152	0.87	(0.71–1.07)	0.18213
40–60	0.81	(0.67–0.99)	0.0422	0.88	(0.71–1.10)	0.26745
>60	1.3	(0.96–1.77)	0.0921	1.17	(0.84–1.63)	0.34707
Sex:						
Female	ref.	ref.	ref.			
Male	1.09	(0.99–1.19)	0.078			
Marital Status:						
Married	ref.	ref.	ref.	ref.	ref.	ref.
Divorced	1.1	(0.95–1.27)	0.187	0.93	(0.80–1.09)	0.36074
Single	1.39	(1.19–1.64)	<0.001	1.17	(0.98–1.39)	0.07681
Widowed	1.28	(1.14–1.45)	<0.001	1.04	(0.92–1.19)	0.52211
Point of entry:						
VCT	ref.	ref.	ref.			
Health Care Facility	0.97	(0.83–1.12)	0.645			
PMTCT	1.06	(0.84–1.35)	0.62			
Other	0.58	(0.22–1.54)	0.27			
Profession:						
Employed	ref.	ref.	ref.	ref.	ref.	ref.
Unemployed	0.8	(0.72–0.89)	<0.001	0.99	(0.88–1.11)	0.87392
Other	0.76	(0.63–0.90)	0.00218	1.03	(0.85–1.24)	0.79496
Year of enrolment:						
2007	ref.	ref.	ref.	ref.	ref.	ref.
2008	0.98	(0.84–1.14)	0.76781	1.37	(1.17–1.59)	< 0.001
2009	0.65	(0.52–0.80)	<0.001	1.24	(1.00–1.54)	0.04843
2010	0.52	(0.44–0.62)	<0.001	1.62	(1.34–1.96)	< 0.001
2011	0.51	(0.43–0.61)	<0.001	1.77	(1.46–2.15)	< 0.001
2012	0.53	(0.44–0.64)	<0.001	1.55	(1.26–1.90)	< 0.001
2013	0.67	(0.55–0.82)	<0.001	2.17	(1.74–2.71)	< 0.001
2014	0.93	(0.76–1.14)	0.49501	4.05	(2.99–5.48)	< 0.001
2015	0.7	(0.56–0.87)	0.00116	2.67	(1.82–3.91)	< 0.001
2016	1.06	(0.82–1.38)	0.64572	4.44	(2.80–7.03)	< 0.001
ART regimen:						
TDF+3TC+EFV	ref.	ref.	ref.	ref.	ref.	ref.
D4T+3TC+EFV	0.82	(0.66–1.02)	0.0763	1.61	(1.18–2.20)	0.00245
D4T+3TC+NVP	0.7	(0.59–0.83)	<0.001	1.58	(1.19–2.09)	0.00156
TDF+3TC+NVP	0.6	(0.47–0.77)	<0.001	0.78	(0.56–1.09)	0.15077
AZT+3TC+NVP	0.64	(0.40–1.02)	0.062	1.59	(0.93–2.71)	0.08858
Other	1.37	(0.83–2.28)	0.2208	2.56	(1.46–4.49)	0.00102
Never initiated	12.93	(11.12–15.03)	<0.001	37.14	(27.97–49.30)	< 0.001
Enrolment CD4 count:						
>200	ref.	ref.	ref.	ref.	ref.	ref.
<10	1.96	(1.59–2.41)	<0.001	2.97	(2.37–3.73)	< 0.001
10 to 50	1.78	(1.54–2.05)	<0.001	2.3	(1.98–2.68)	< 0.001
50–100	1.18	(1.01–1.37)	0.0336	1.58	(1.35–1.85)	< 0.001
100–200	0.88	(0.77–1.01)	0.0744	1.48	(1.27–1.71)	< 0.001
Tuberculosis diagnosis:						
Not diagnosed	ref.	ref.	ref.	ref.	ref.	ref.
Within first 2 months	0.48	(0.41–0.55)	<0.001	1.18	(0.96–1.45)	0.12336
Between 2 to 6 months	0.49	(0.36–0.68)	<0.001	1.15	(0.79–1.66)	0.47636
Between 6 to 12 months	0.22	(0.12–0.41)	<0.001	0.64	(0.33–1.25)	0.19345

Cox proportional hazards regression analysis for risk of attrition in those with advanced HIV disease (Lost to follow up and death) over a period of 12 months of follow up. Complete case analysis approach used (n = 7260). Those variables not significant in the univariate analysis were dropped from the multivariate model.

Drug abbreviations; D4T: Stavudine, 3TC: Lamivudine, EFV: Efavirenz, NVP: Nevirapine, AZT: Zidovudine, TDF: Tenofovir.

In February 2015 differentiated care for patients presenting with advanced HIV disease and a CD4 count less than 100 cells/mm^3^ was introduced, a total of 377 patients enrolled between February 2015 and end of June 2016 were eligible for this differentiated care. We compared the outcomes of these patients with those who were enrolled with a CD4 count below 100 cells/mm^3^ stage in a period between 2010 and end of January 2015, these dates were chosen in order for there to be a similar median time to ART initiation between groups (median time to initiation of ART was 21 days in 2010–15 and 15 days in 2015–16, Fig 2C).

**Fig 3:**
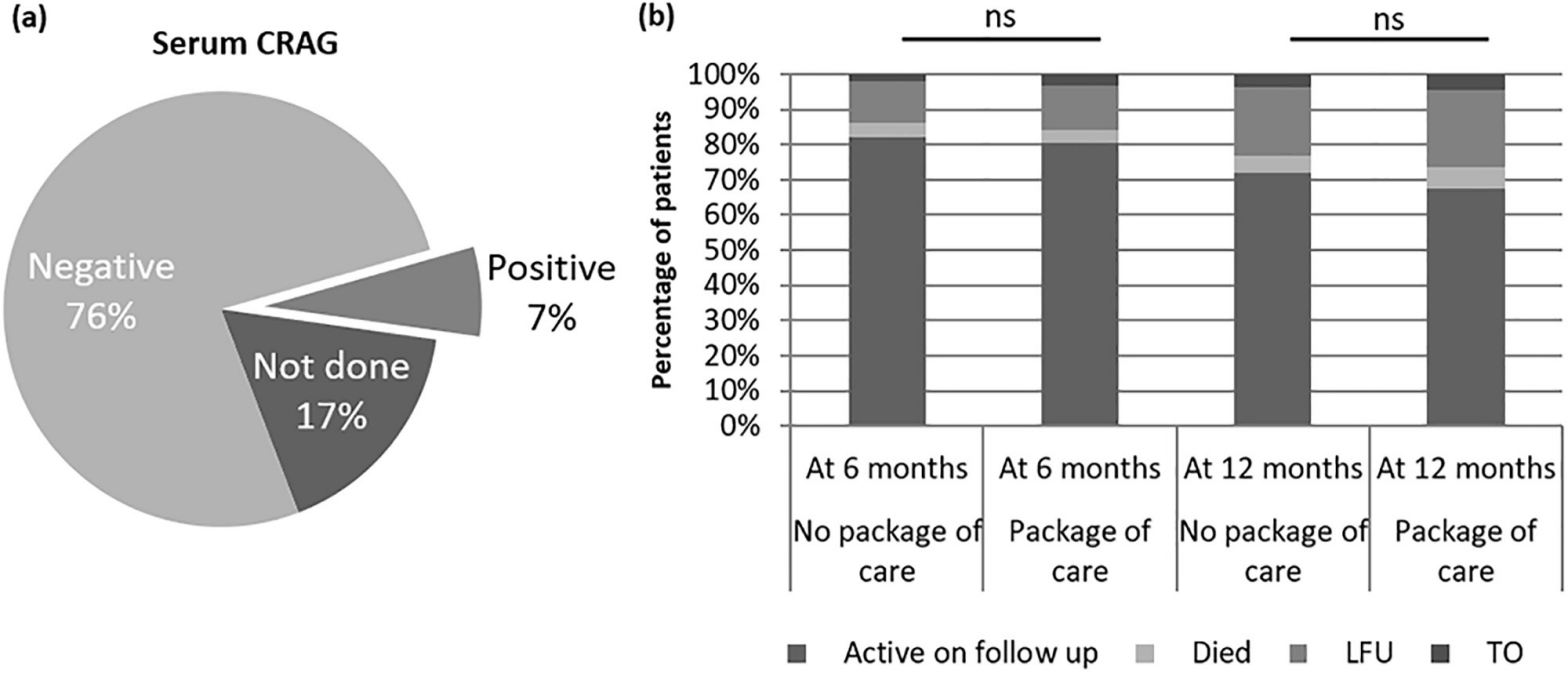
Package of care for very late presenters. **(A)** Pie chart showing results of Serum cryptococcal antigen (CRAG) testing in the 377 patients eligible for the package of care for very advanced disease between February 2015 and end June 2016. **(B)** Treatment outcomes at 6 months and 12 months for patients presenting for first visit with very advanced disease (CD4 count less than 100 cells/mm^3^). Very advanced disease patients receiving no package of care patients enrolled between 2010 and End January 2015 (n = 1911), Package of care patients enrolled between February 2015 and end June 2016 (n = 377). Statistical test, Chi-Square, ns = non-significant p>0.05).

Serum CRAG testing was positive in 7% of patients of the 377 patients eligible for testing (Fig 3A), in those who had a positive serum CRAG and went on to have a CSF CRAG it was positive in 44% of patients (S1 Table). In those who had a negative serum CRAG there was no incidence of a positive CSF CRAG in the limited numbers of patients in whom it was done in (n = 8, S1 Table). Routine screening for tuberculosis was also undertaken by sputum screening with GeneXpert resulting in a significant increase in those diagnosed and treated for TB, 35.3% of patients with advanced HIV receiving the differentiated care were diagnosed with TB compared to 26.8% in those with advanced HIV in the period previous to the introduction, this increase in diagnosis was within 2 months of enrollment to the clinic (S2 Table). Two patients were identified to be both serum CRAG positive and co-infected with TB at first presentation.

There was no significant difference in outcome between those receiving the differentiated care compared to those who did not receive it in the period before, with retention in the cohort at 12 months 72.1% in the period before the differentiated care and 67.4% during the period that differentiated care was available (no significant difference, Fig 3B).

## Conclusions

We report here the outcomes for a large cohort of HIV patients undergoing care at a semi-urban polyclinic on the outskirts of Harare, Zimbabwe. Over the course of the study the percentage of patients enrolling with advanced HIV disease significantly reduced although it still remained high by the end of the study period at 40% of ART naïve patients enrolling with advanced HIV disease.

A number of patients were excluded from the study (2484 patients) due to the lack of CD4 count or WHO stage preventing classification at time of enrolment into the cohort, these missing patients affect the completeness of the analysis and may therefore limit the interpretation of the data. All efforts were made to make the data as complete as possible, however one of the limitations of retrospective cohort analyses, particularly those spanning over a prolonged time period include variations in data capture and completeness.

In our study the median CD4 count at first visit increased by a modest 4.8 cells/mm^3^ per year, other studies have shown either similarly modest or no significant increase in CD4 counts at presentation over time [8,22], in keeping with this and similar to other studies the proportion of patients presenting with advanced HIV disease decreased significantly overtime [23]. This together with changes in ART eligibility criteria impacted on the median time to initiation on ART over time, with the median time to initiation falling over the course of the study as has also been experienced in other settings [24,25].

Between 2007 and 2009 the median time to initiation on ART even for those eligible (those with advanced HIV disease) was above 100 days and this was primarily due to a policy implemented at the clinic level which required at least 2 counselling sessions conducted a period of time apart to have been undertaken prior to initiation on ART. This policy was implemented to ensure that patients were likely to adhere and continue on ART which was in limited supply at the time. At other times entry into the cohort was restricted due to limitations on the availability of ART. During some of these times women and children were prioritized for initiation and enrollment into the cohort restricted during such as in 2009 when an ongoing political and economic crisis within the country limited the availability of ART nationwide [26]. Once these restrictions were removed and following changes in national guidance, the median time to initiation in those eligible for ART at first visit has fallen to less than 15 days and with the advent of test and treat in Zimbabwe from December 2016 this time to initiation and pre-ART attrition should reduce further [27]. These additional restrictions on enrollment for ART may have affected the enrollment into the cohort, with those ineligible not always enrolled into the cohort and therefore the percentage of patients enrolled with advanced HIV disease may be overrepresented compared to those tested in the early periods of the study when these additional restrictions were in place.

Late presentation of HIV disease has been associated with male sex, stigma, increasing age, low socio-economic status, poor social support and fear of stigma [6,9–12,28,29]. Similar to these studies we identified male sex, older age, referral from a health care facility and not being married as being significant risk factors for late presentation in our study. It may be that the proportion of patients presenting with advanced disease is overestimated in the dataset as earlier during the study period those patients with early disease may not have been registered in the database due to the restrictive criteria surrounding initiation on ART. Patients presenting with advanced HIV disease were at increased risk of attrition at 12 months if they were single and the lower the first visit CD4 cell count at first presentation. Regimens containing Stavudine were also at increased risk of attrition, regimens containing this drug have already been phased out of use in Zimbabwe.

Management of patients with advanced HIV disease is complex and requires prompt diagnosis of any potential opportunistic infections [13]. Following the introduction of differentiated care for patients with advanced HIV disease, cryptococcalaemia was detected in 7% of eligible patients and there was a significant increase in the detection and treatment of TB. Despite this increased detection of opportunistic infections there was no significant reduction in rates of attrition at 6 or 12 months for patients receiving this package of differentiated care compared to those who did not receive it in the period before.

The implementation of the differentiated care for patients with advanced HIV disease faced challenges in the Epworth polyclinic. Often, not all patients that were eligible received the entire package of differentiated care as referrals were occasionally missed by the clinic staff and those whose point of entry was via the TB service could bypass the service. The model of differentiated care provided in this study may have benefited from the addition of other potential components included in the recently published WHO guidance such as urinary lipoarabinomannan testing which can be of importance in identifying TB infection in those with advanced HIV disease[13].

The Epworth polyclinic also had organizational changes during the study period including a reduction in the size of the community defaulter tracer team which may have resulted in increased attrition rates and the significantly increased risk of attrition over 12 months of follow up seen in patients with advanced HIV disease from 2013 onwards in the cox-proportionate hazard analysis. These organizational changes may have masked any potential impact of the model of differentiated care for patients with advanced HIV disease.

This study emphasizes that presentation with advanced HIV disease continues to be a significant burden in this setting. Patients with advanced HIV disease have significantly worse outcomes with higher recorded mortality compared to those presenting at an earlier stage. The introduction of differentiated care for such patients resulted in increased diagnosis and treatment of opportunistic infections and emphasizes the need for the introduction of routine screening for such infections in this population.

## Supporting information

S1 Table Results of serum and CSF cryptococcal antigen testing. Table of cryptococcal antigen testing undertaken in patients with a CD4 count of less than 100 cells/mm^3^ at first visit between February 1^st^ 2015 and end June 2016. CSF: Cerebrospinal fluid.
(DOCX)

S2 Table Diagnosis of tuberculosis after enrollment into cohort. Timing of tuberculosis diagnosis following enrollment in to the cohort. Diagnosis made either by GeneXpert, sputum smear, chest x-ray or compatible clinical symptoms and/or signs. Advanced HIV disease patients those patients enrolled at first visit with a CD4 count less than 200 cells/mm3or WHO stage 3 or 4. Early patients those with a CD4 count greater than 200 cells/mm3and WHO stage 1 or 2. Very advanced disease patients (CD4 count less than 100 cells/mm3) receiving no package of care patients enrolled between 2010 and End January 2015 (“no package”, n = 1911), Very advanced disease patients (CD4 count less than 100 cells/mm3) receiving differentiated care including sputum screening enrolled between February 2015 and end June 2016 (“Package”, n = 377). Statistical test between groups: Chi-Square.
(DOCX)
