# Supplementary material for: Lessons learned: Retrospective assessment of outcomes and management of patients with advanced HIV disease in a semi-urban polyclinic in Epworth, Zimbabwe
Source: PLoS One. 2019 Apr 10;14(4):e0214739. doi: 10.1371/journal.pone.0214739 (PMC6457534; doi:10.1371/journal.pone.0214739)
Supplement: S1 Table — Table of cryptococcal antigen testing undertaken in patients with a CD4 count of less than 100 cells/mm3 at first visit between February 1st 2015 and end June 2016. CSF: Cerebrospinal fluid. (DOCX) [file pone.0214739.s001.docx]

|  |  | Serum | | |
| --- | --- | --- | --- | --- |
|  |  | Not done | Negative | Positive |
| CSF | Not done | 64 | 280 | 7 |
|  | Negative | 0 | 8 | 10 |
|  | Positive | 0 | 0 | 8 |

**Supplementary Table 1: Results of serum and CSF cryptococcal antigen testing**

Table of cryptococcal antigen testing undertaken in patients with a CD4 count of less than 100 cells/mm^3^ at first visit between February 1^st^ 2015 and end June 2016. CSF: Cerebrospinal fluid.
