# Supplementary material for: Lessons learned: Retrospective assessment of outcomes and management of patients with advanced HIV disease in a semi-urban polyclinic in Epworth, Zimbabwe
Source: PLoS One. 2019 Apr 10;14(4):e0214739. doi: 10.1371/journal.pone.0214739 (PMC6457534; doi:10.1371/journal.pone.0214739)
Supplement: S2 Table — Timing of tuberculosis diagnosis following enrollment in to the cohort. Diagnosis made either by GeneXpert, sputum smear, chest x-ray or compatible clinical symptoms and/or signs. Advanced HIV disease patients those patients enrolled at first visit with a CD4 count less than 200 cells/mm3or WHO stage 3 or 4. Early patients those with a CD4 count greater than 200 cells/mm3and WHO stage 1 or 2. Very advanced disease patients (CD4 count less than 100 cells/mm3) receiving no package of care patients enrolled between 2010 and End January 2015 (“no package”, n = 1911), Very advanced disease patients (CD4 count less than 100 cells/mm3) receiving differentiated care including sputum screening enrolled between February 2015 and end June 2016 (“Package”, n = 377). Statistical test between groups: Chi-Square. (DOCX) [file pone.0214739.s002.docx]

|  | Overall  n=16007 | Early  n=8420 | Advanced  n=7587 | p value | No Package  n=1991 | Package  n=377 | p value |
| --- | --- | --- | --- | --- | --- | --- | --- |
| Timing of TB diagnosis |  |  |  | <0.001 |  |  | 0.006 |
| Not diagnosed | 13906 (86.9%) | 8142 (96.7%) | 5764 (76.0%) |  | 1399 (73.2%) | 244 (64.7%) |  |
| Within 2 months | 1537 (9.60%) | 112 (1.33%) | 1425 (18.8%) |  | 448 (23.4%) | 114 (30.2%) |  |
| 2 to 6 months | 331 (2.07%) | 74 (0.88%) | 257 (3.39%) |  | 48 (2.51%) | 13 (3.45%) |  |
| 6 to 12 months | 233 (1.46%) | 92 (1.09%) | 141 (1.86%) |  | 16 (0.84%) | 6 (1.59%) |  |

**Supplementary Table 2: Diagnosis of tuberculosis after enrollment into cohort**

Timing of tuberculosis diagnosis following enrollment in to the cohort. Diagnosis made either by GeneXpert, sputum smear, chest x-ray or compatible clinical symptoms and/or signs. Advanced HIV disease patients those patients enrolled at first visit with a CD4 count less than 200 cells/mm3or WHO stage 3 or 4. Early patients those with a CD4 count greater than 200 cells/mm3and WHO stage 1 or 2. Very advanced disease patients (CD4 count less than 100 cells/mm3) receiving no package of care patients enrolled between 2010 and End January 2015 (“no package”, n=1911), Very advanced disease patients (CD4 count less than 100 cells/mm3) receiving differentiated care including sputum screening enrolled between February 2015 and end June 2016 (“Package”, n=377). Statistical test between groups: Chi-Square.
